# Der schnellende Finger – Pitfalls und Differenzialdiagnostik

**DOI:** 10.1007/s00132-023-04390-6

**Published:** 2023-05-26

**Authors:** A. Cavalcanti Kußmaul, A. Ayache, F. Unglaub

**Affiliations:** 1 Klinik für Orthopädie und Unfallchirurgie, Muskuloskelettales Universitätszentrum München (MUM), Klinikum der Universität München, LMU München, Marchioninistr. 15, 81377 München, Deutschland; 2 Handchirurgie, Vulpius Klinik, Bad Rappenau, Deutschland; 3 Medizinische Fakultät Mannheim, Universität Heidelberg, Heidelberg, Deutschland

**Keywords:** Exostose, Bänder, Gelenk, Metakarpophalangealgelenk, Schnellender Finger, Stenosierende Tendovaginitis, Exostosis, Ligaments, articular, Metacarpophalangeal joint, Snapping finger, Stenosing tendovaginitis

## Abstract

Die Differenzialdiagnostik des schnellenden Fingers stellt eine klinische Herausforderung dar. Im vorgestellten Fall wird ein 32-jähriger Patient präsentiert, welcher sich mit einem persistierenden Schnappen des rechten Zeigefingers im Metakarpophalangealgelenk ohne lokale Druckschmerzhaftigkeit trotz vorheriger operativer A1-Ringbandspaltung vorstellte. In der CT-Diagnostik konnte ein prominentes Tuberculum articularis nachgewiesen werden. Die MRT-Bildgebung war unauffällig. Durch operative Revision mit begleitender Abtragung des Tuberkulums ließ sich eine volle Funktion des Zeigefingers wieder herstellen.

**Video online:**

Die Online-Version dieses Beitrags (10.1007/s00132-023-04390-6) enthält Video 1 und Video 2.

## Abstract (en)

The differential diagnosis of a trigger finger presents a clinical challenge. This case depicts a 32-year-old male patient who presented with persistent snapping of the right index finger at the metacarpophalangeal joint without localized tenderness despite previous surgical A1-annular ligament release. CT diagnostics demonstrated a prominent articular tuberosity. The MRI showed no pathological findings. Surgical revision with concomitant excision of the tuberosity restored smooth mobility of the index finger.

## Anamnese

Ein 32-jähriger männlicher Patient stellte sich mit seit Sommer 2022 bestehendem, spontan aufgetretenem, schmerzlosem „Einschnappen“ mit anschließender schmerzhafter Reposition des rechten Zeigefingers im Grundgelenk in unserer handchirurgischen Ambulanz vor. Extern habe im Vorfeld klinisch der Verdacht auf eine Ringbandstenose bestanden, woraufhin im August 2022 eine A1-Ringbandspaltung durchgeführt worden war. Postoperativ habe das „Schnappen“ jedoch persistiert.

## Befund

Inspektorisch zeigten sich reizlose Wundverhältnisse ohne ausgeprägte Narbenbildung bei prominentem radialseitigem Kopf des zweiten Mittelhandknochens (MHK II) rechts. Palpatorisch bestand kein lokaler Druckschmerz über dem A1-Ringband bei Reproduzierbarkeit der inspektorischen Prominenz. Ein Faustschluss sowie die anschließende Streckung der Finger waren ohne Schnappen möglich. Bei Flexion des rechten Zeigefingers im Metakarpophalangealgelenk (MKP) und gleichzeitiger Ulnarduktion konnte ein „Einhaken“ des Zeigefingers provoziert werden, welches sich durch aktive oder passive Extension im MKP-Gelenk mit unangenehmen „Schnappen“ lösen ließ.

## Diagnose

**Figure 1:**
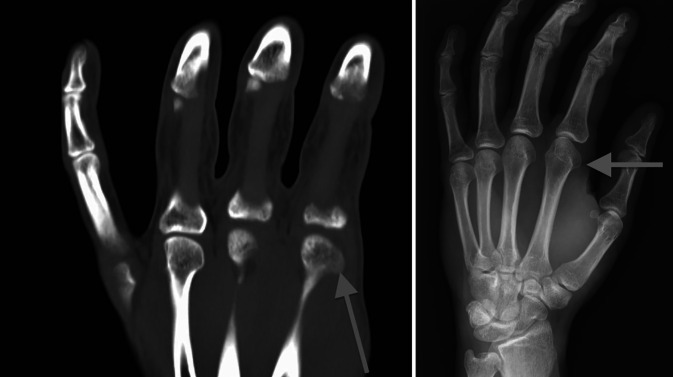

Aufgrund des untypischen Befundes nach operativer Ringbandspaltung und Verdacht auf knöcherne, artikuläre Inkongruenz erfolgte zunächst die Durchführung eines Röntgenbildes, welchem sich die Durchführung einer Dünnschicht-Computertomographie (CT) und einer Magnetresonanztomographie (MRT) anschloss. Sowohl im initialen Röntgen als auch in der CT zeigte sich ein prominentes Tuberculum articularis radialseitig entsprechend eines „boxers knuckle“ (Abb. 1) bei unauffälliger MRT.

## Therapie und Verlauf

**Figure 2:**
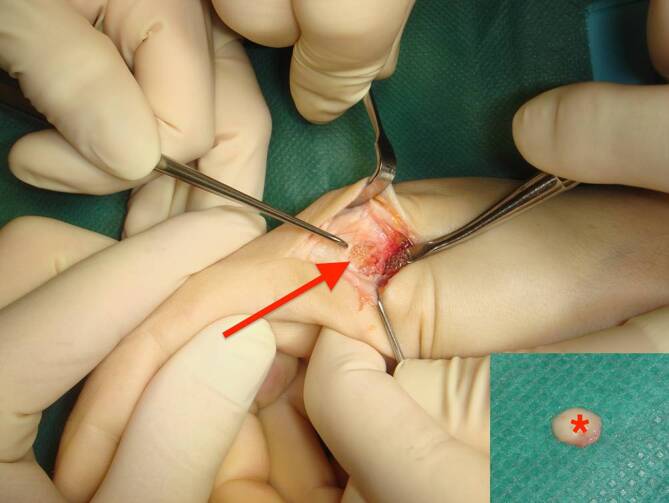

Nach ausführlicher Aufklärung wurde die Indikation zur operativen Revision gestellt. Diese erfolgte im November 2022 in Plexusanästhesie und Oberarmblutleere. Nach S‑förmiger Inzision über dem radialseitigen MKP und penibler Gelenkpräparation fand sich ein ausgeprägtes Tuberculum articularis, welches mit dem Meißel abgetragen wurde (Abb. 2). Intraoperativ bestand kein Anhalt auf eine Instabilität, sodass auf eine Reinsertion der Seitenbandstrukturen verzichtet werden konnte. Postoperativ sistierte das Schnappen bei uneingeschränkter, schmerzfreier Mobilität.

## Diskussion

Der schnellende Finger (Tendovaginitis stenosans) ist mit einer Lebenszeitprävalenz von 2–3 % die häufigste Beeinträchtigung der Hand [1]. Am häufigsten betroffen sind der Mittel- und Ringfinger sowie die weibliche Bevölkerung [1]. Ursächlich ist in der Regel eine Verdickung des A1-Ringbandes oder der darunter verlaufenden Beugesehne, welche durch die entstehende lokale Stenose das Gleiten der Sehne im Bereich des A1-Ringbandes stört [1, 2].

Die Diagnose des schnellenden Fingers wird meist klinisch gestellt: Patienten klagen über eine lokale, palmarseitige Druckschmerzhaftigkeit über dem A1-Ringband und ein „Schnappen“ im Bereich des MKP des betreffenden Fingers bei Extension aus dem Faustschluss, welches das Greifen und Halten von Gegenständen erschweren kann [2]. Mittels Sonographie kann eine Sicherung der Diagnose durch Darstellung der verdickten Strukturen erfolgen [3]. Therapeutisch kommen bei milden Verläufen Analgesie, ggf. kurze Ruhigstellung sowie Kortikosteroidinjektion in Betracht [2]. Bei frustraner konservativer Therapie ist eine operative Therapie mittels A1-Ringbandspaltung indiziert, welche Erfolgsraten von bis zu 90 % bei Komplikationsraten von ca. 5–12 % aufweist [4–6].

Differenzialdiagnostisch kommen Osteophyten am Metakarpalekopf, freie Gelenkkörper, eine Dislokation der palmaren Platte, ein „boxers knuckle“ sowie selten eine (sub-)luxierte Strecksehnenaponeurose in Betracht [7]. Da es sich hier dann um Fälle mit „untypischer Klinik“, vor allem fehlenden Schmerzen über dem A1-Ringband sowie untypischem Schnappen, handelt, sollte eine differenzierte klinische Untersuchung, auch von dorsal, durchgeführt und die radiologische Diagnostik „ausgereizt“ werden: Während eine Dünnschicht-CT dem Nachweis knöcherner Veränderungen dient, kann eine MRT weichteilige Pathologien detektieren.

Ein „boxers knuckle“, wie in dem hier beschriebenen Fall, betrifft vor allem junge Patienten bei zugrunde liegenden knöchernen Prominenzen am radialen Kondylus [7, 8].

Grundsätzlich sind die MKP anatomisch ellipsoid aufgebaut [9]. Als metakarpalseitige Überreste einer wahrscheinlich ehemals bikondylären Gelenkfläche finden sich am Zeigefinger radial und am Kleinfinger ulnar die besonders prominenten Tubercula articularia. Hierdurch können normalerweise die hier verlaufenden Muskelstränge wie über ein Hypomochlion hinwegziehen [9]. In Flexion artikuliert das MKP primär radialseitig, wohingegen in Extension eine eher ulnarseitige Artikulation besteht [9]. Pathophysiologisch kann es entsprechend bei Hypertrophie oder knöcherner Exostose im Bereich des radialen Kondylus des zweiten Fingers zu einer Subluxation des Gelenks bei Flexion mit oder ohne begleitendes Fixieren von Seitenbandstrukturen, was letztlich auch zu einer fixierten Blockade führen kann, kommen [7].

In dem vorliegenden Fall kann die artikuläre Subluxation mit entsprechendem Verhaken wie folgt erklärt werden: Der oben beschriebene vermehrte radialseitige Gelenkkontakt in Flexion führte zu einer ulnarseitigen Achsabweichung des Fingergrundglieds des Zeigefingers. Bei additivem Stress in Ulnarduktion kommt es schließlich zu einem Abrutschen der Kollateralbandstrukturen und zu einem Verhaken palmarseitig des Tuberkulums mit einer Fixierung des Grundgelenks (Video 1).

Abschließend sollte sich die Therapie nach der zugrundeliegenden Pathologie richten. Da es sich bei diesem Fall um eine mechanische Ursache handelte, konnte durch eine operative Abtragung derselben Beschwerdefreiheit erzielt werden (Video 2). Die „wide awake local anesthesia no tourniquet“(WALANT)-Technik bietet hier zwar den Vorteil der intraoperativen aktiven Funktionsprüfung, jedoch kann das „Aufquellen“ des Operationsgebietes durch die Lokalanästhesie zu erschwerten operativen Bedingungen führen [10]. Alternativ käme hier ein distaler Handblock infrage, bei dem zusätzlich zur Oberarmblutleere eine Lokalanästhesie appliziert wird [11].

## Fazit für die Praxis

- Nicht jedem schnellenden Finger liegt eine Tendovaginitis stenosans zugrunde.
- Bei untypischem klinischem Befund sollte eine differenzierte klinische Untersuchung mit anschließender adäquater Bildgebung erfolgen, um differenzialdiagnostische Ursachen zu detektieren und entsprechend zu behandeln.

## Supplementary Information





